# Prevalence of and Susceptibility to Cigarette Smoking Among Female Students Aged 13 to 15 Years in Vietnam, 2007

**Published:** 2009-12-15

**Authors:** Hoang Van Minh, Phan Thi Hai, Ly Ngoc Kinh, Kim Bao Giang

**Affiliations:** Faculty of Public Health, Hanoi Medical University Vietnam; Vietnam Steering Committee on Smoking and Health, Hanoi, Vietnam; Vietnam Steering Committee on Smoking and Health, Hanoi, Vietnam; Hanoi Medical University Vietnam, Hanoi, Vietnam

## Abstract

**Introduction:**

Recent reports show a sharp increase in smoking rates among girls. We describe prevalence of cigarette smoking and susceptibility to cigarette smoking among female students aged 13 to 15 years in Vietnam and examine the associated factors.

**Methods:**

We used data from female secondary school students aged 13 to 15 years (grades 8-10) from the 2007 Global Youth Tobacco Survey that was conducted in 9 provinces in Vietnam. We used multivariate logistic regression analysis to determine associations between independent variables with smoking status and susceptibility to smoking.

**Results:**

Prevalence of cigarette smoking among girls was 1.2% (95% confidence interval [CI], 0.9-1.5), and 1.5% (95% CI, 1.2-1.9) of girls were susceptible to smoking. Having friends who smoke was the strongest predictor of both smoking status and susceptibility to smoking. Attendance at school classes that described the harmful effects of smoking had significant effects in reducing cigarette smoking. Girls who were exposed to billboard cigarette advertising were more likely to be susceptible to smoking than were those who had not seen advertisements.

**Conclusion:**

Our findings highlight the need for pursuing school-based intervention programs in Vietnam and for countering tobacco advertising and marketing practices that target young women.

## Introduction

Tobacco use is one of the leading causes of illness and death in the world (1,2). Tobacco use is associated with many chronic diseases, such as cardiovascular disease, cancer, chronic respiratory diseases, and diseases of the digestive tract (1,2). Although the prevalence of smoking among women is lower than among men worldwide (42% of men and 24% of women in developed countries and 48% of men and 7% of women in developing countries) (3), the issue of smoking among women has attracted growing attention because recent reports have shown a high level of tobacco use among girls (4,5). Furthermore, women face additional health risks, including hazards during pregnancy (eg, exposure to secondhand smoke that can lead to low birth weight or premature birth) and cancers of the female reproductive system (eg, cancer of the cervix) (4,6). Smoking also impoverishes many women by negatively affecting their health (7).

Many female smokers started smoking as adolescents, and girls have more difficulty than do boys quitting smoking because they experience stronger dependence on the behavior and more negative emotions during attempts to quit (8). More concern now exists regarding smoking among girls because the tobacco industry has been extensively investing in advertising and marketing campaigns that associate tobacco use with independence, desirable body image, glamour, and romance, and these campaigns target girls and women (4,9,10).

In Vietnam, the prevalence of smoking among girls in 2003 was approximately 1.5% (11). However, like other developing countries in Asia, Vietnam is affected by tobacco companies' girl-specific cigarette-promoting campaigns (10). Vietnam has made progress in banning cigarette advertisements in mass media; however, because of the tactics used by the tobacco industry and because of the weak enforcement of the law, cigarette promotion (eg, point of sale, billboard advertisements, offering objects with a cigarette brand logo, tobacco company representatives giving out free cigarettes) is still commonly observed. A recent study from Vietnam showed that more than 25% of girls aged 13 to 25 years had ever noticed things that encouraged them to smoke in the past 30 days (12). Problems associated with smoking in Asia among women and girls will continue to be evident until well into the 21st century if no action is taken now to curb the smoking epidemic (4).

By using data collected from the 2007 Global Youth Tobacco Survey (GYTS) in Vietnam, we describe the prevalence of and susceptibility to cigarette smoking among female students aged 13 to 15 years in Vietnam and examine their associated factors. Our aim is to provide information for designing appropriate preventive strategies.

## Methods

The 2007 GYTS was conducted in 9 provinces in Vietnam (North: Ha noi, Hai phong, Hoa binh, Tuyen Quang; Center: Hue, Da nang; South: Ho Chi Minh City, Lam dong, Tien giang). Participants of this study were secondary school students aged 13 to 15 years (grades 8-10). The GYTS is a school-based survey of a defined geographic site and uses a standardized method for constructing sampling frames, selecting schools and classes, preparing questionnaires, conducting field procedures, and processing data. A 2-stage cluster-sample design was used to produce representative data for Vietnam. During the first stage, schools were selected with probability proportional to enrollment size. During the second stage, classes were randomly selected, and all students in selected classes were eligible to participate. The survey questionnaire was adapted from a standardized version developed by the Centers for Disease Control and Prevention and the World Health Organization. Data were collected by field staff from 9 provinces. During a 2-day training period, data collectors were trained to do the sampling (choose classes) and administer the questionnaires in a standardized way (13).

Smoking status and susceptibility to smoking among schoolgirls were the dependent variables (Table 1). Three questions were asked to evaluate susceptibility to smoking among study respondents: 1) "If 1 of your best friends offered you a cigarette, would you smoke it?", 2) "At any time during the next 12 months do you think you will smoke a cigarette?", and 3) "Do you think you will be smoking cigarettes 5 years from now?" Possible responses were "definitely not," "probably not," "probably yes," and "definitely yes." Students who had never smoked and answered "definitely not" to all 3 questions were considered to be nonsusceptible; all other students were considered to be susceptible. Independent variables were age, perception of smoking habit, knowledge of harmful effects of smoking, parents' smoking status, friends' smoking status, education on harmful effects of smoking, access to antismoking media, and exposure to billboard cigarette advertising.

Data were analyzed by using Stata version 10 (StataCorp LP, College Station, Texas). We used multivariate logistic regression analysis to determine associations between smoking status and susceptibility to smoking and other relevant variables. All data were weighted to represent the total school population in Vietnam. The Ministry of Health of Vietnam approved this study.

## Results

A total of 8,391 female students aged 13 to 15 years participated in the 2007 GYTS in Vietnam. Of these, 7,967 (95%) responded to all the study questions. There were similar proportions of study participants in each age group (Table 2). More than half (55.0%) of study participants had either a father or mother who smoked, and 2% of participants reported having both a father and mother who smoked. Approximately 38% of study participants reported having close friends who smoke. Prevalence of current cigarette smoking among girls aged 13 to 15 years was 1.2%, and 1.5% of study participants were susceptible to smoking (Table 3). Current cigarette smoking was significantly higher among girls aged 15 years than among younger girls.

Girls who said that smoking cigarettes helps people feel more comfortable were twice as likely to be current smokers as girls who did not feel this way (Table 4). Girls who thought smoking cigarettes makes girls look more attractive were significantly more likely to be current smokers and to be susceptible to smoking than girls who thought smoking cigarettes makes girls look less attractive or thought it made no difference. Participants with close friends who smoke were more than 6 times as likely to smoke and more than 3 times as likely to be susceptible to smoking than participants whose close friends did not smoke.

Exposure to billboard cigarette advertising increased the odds both of being a current smoker and of being susceptible to smoking, but only significantly for being susceptible to smoking. Good knowledge of the harmful effects of secondhand smoke was associated with significant reductions in the odds of being a current smoker and of being susceptible to smoking. Girls who attended classes about the harmful effects of smoking were less likely to be current smokers than girls who did not attend. Girls who had access to antismoking media were less likely to be susceptible to smoking than girls who did not have access.

## Discussion

We found a lower prevalence of cigarette smoking among girls aged 13 to 15 years in Vietnam than did the 2003 GYTS in Vietnam (1.5%) (11). We also found a lower prevalence than was found among girls for the whole Western Pacific region from 1999 through 2005 (3.3%) (14). Our findings suggest that Vietnam may have been successful in preventing and controlling cigarette smoking and tobacco use among schoolgirls. However, continued efforts are needed to maintain and promote this initial success.

We found that 1.5% of never-smoking girls were susceptible to smoking. Susceptibility to smoking is a public health concern because it is a significant predictor of smoking initiation and addiction (15-18). Many adult smokers started smoking as adolescents or young adults. Furthermore, susceptibility to smoking is amenable to interventions (19). A comprehensive intervention against tobacco use is needed to break the pathway from susceptibility to smoking initiation.

Our findings showed that having friends who smoke was the strongest predictor of both smoking status and susceptibility to smoking. Previous international studies also revealed a strong association between peers who smoke and smoking status (20,21). In Vietnam, the bond of friendship is a part of the adolescent development process, and peer pressure is one of the most common reasons that young people start smoking. Maintaining a smoke-free school environment would reduce the likelihood that any student will start smoking. School environment has been found to influence susceptibility to smoking among school students in Laos, Cambodia, and Vietnam (22).

Contrary to findings that suggest that school-based smoking-prevention programs do not have long-term success at keeping youths from starting to smoke (23), we found that implementing school classes that explain the harmful effects of smoking was associated with a significant reduction in cigarette smoking prevalence among girls who attended the classes. This finding highlights the need for establishing school-based intervention programs in Vietnam. Programs should also offer information about problems unique to women (eg, cancers of the female reproductive system). A curriculum containing information about the harmful effects of smoking has been found to be effective in changing children's attitudes toward smoking; the main predictors of intention to smoke were attitude and refusal skills (24). Another school-based curriculum for adolescent smoking cessation has had a measurable effect on the smoking habits of participants (25).

We found that girls exposed to billboard cigarette advertising were more likely to be susceptible to smoking than girls who had not seen advertisements. International studies suggest that exposure to tobacco advertising and promotion is associated with the likelihood that adolescents will start to smoke (26). Our finding underscores the need to counter the tobacco advertising and marketing practices that target young women.

Our study has several limitations. First, data from the GYTS are self-reported. Although Brener et al found highly reliable results on teenage smoking when data were self-reported (27), accuracy of reporting is not known in the Vietnamese setting. Second, the GYTS was administered to youths who were in school on the day of the survey and who completed the survey; therefore, findings may be conservative. Third, the GYTS is limited to youths who attend school and did not include youths who were outside the school environment. Fourth, some variables, such as antismoking media messages and billboard cigarette advertising (especially if these variables are not school averages), may be not independent of girls' susceptibility or smoking status. Finally, the predictive validity of susceptibility to smoking is unknown in low-income and middle-income countries.

Little is known about smoking among girls in Vietnam. We provide evidence on the issue of smoking among young female students in Vietnam and its correlates. Findings from our study can be used to address cigarette smoking among young girls in Vietnam and other countries.

## Figures and Tables

**Table 1:** Description of Study Variables, Global Youth Tobacco Survey, Vietnam, 2007

Variable	Question	Original Response Option	Definition Used in This Article
**Dependent variables**			
Current cigarette smoking	During the past 30 days (1 month), on how many days did you smoke cigarettes?	1 = 0 day 2 = 1-2 days 3 = 3-5 days 4 = 6-9 days 5 = 10-19 days 6 = 20-29 days 7 = All 30 days	0 = No (response option 1) 1 = Yes (response options 2-7)
Susceptibility to smoking	If 1 of your best friends offered you a cigarette, would you smoke it?	1 = Definitely not 2 = Probably not 3 = Probably yes 4 = Definitely yes	Students who had never smoked: 0 = answered "definitely not" to all 3 questions (nonsusceptible) 1 = all other students (susceptible)
	At any time during the next 12 months, do you think you will smoke a cigarette?	1 = Definitely not 2 = Probably not 3 = Probably yes 4 = Definitely yes	
	Do you think you will be smoking cigarettes 5 years from now?	1 = Definitely not 2 = Probably not 3 = Probably yes 4 = Definitely yes	
**Independent variables**			
Age	How old are you?	1 = Aged 13 y 2 = Aged 14 y 3 = Aged 15 y	1 = Aged 13 y 2 = Aged 14 y 3 = Aged 15 y
Perception of smoking habit	Do you think girls who smoke cigarettes have more or less friends?	1 = More friends 2 = Less friends 3 = No difference from nonsmokers	1 = More friends 2 = Less friends or no difference from nonsmokers
	Does smoking cigarettes help people feel more or less comfortable at celebrations, parties, or in social gatherings?	1 = More comfortable 2 = Less comfortable 3 = No difference from nonsmokers	1 = More comfortable 2 = Less comfortable or no difference from nonsmokers
	Do you think smoking cigarettes makes girls look more or less attractive?	1 = More attractive 2 = Less attractive 3 = No difference from nonsmokers	1 = More attractive 2 = Less attractive or no difference from nonsmokers
Knowledge of harmful effects of smoking	Do you think cigarette smoking is harmful to your health?	1 = Definitely not 2 = Probably not 3 = Probably yes 4 = Definitely yes	1 = Good knowledge (response option 4) 0 = Poor knowledge (response options 1-3)
	Do you think the smoke from other people's cigarettes is harmful to you?	1 = Definitely not 2 = Probably not 3 = Probably yes 4 = Definitely yes	1 = Good knowledge (response option 4) 0 = Poor knowledge (response options 1-3)
Parents' smoking status	Do your parents smoke?	1 = None 2 = Both 3 = Father 4 = Mother	1 = None 2 = Either father or mother 3 = Both parents
Friends' smoking status	Do any of your closest friends smoke cigarettes?	1 = None 2 = Some of them 3 = Most of them 4 = All of them	1 = None 2 = Yes
Education on harmful effects of smoking	During this school year, were you taught in any of your classes about the dangers of smoking?	1 = Yes 2 = No 3 = Not sure	1 = Yes 2 = No or not sure
Access to antismoking media	During the past 30 days (1 month), how many anti-smoking media messages (eg, television, radio billboards, posters, newspapers, magazines, movies, drama) have you seen?	1 = A lot 2 = A few 3 = None	1 = A lot 2 = A few or none
Exposure to billboard cigarette advertising	During the past 30 days (1 month), how many advertisements for cigarettes have you seen on billboards?	1 = A lot 2 = A few 3 = None	1 = A lot 2 = A few or none

**Table 2:** Characteristics of Female Students (N = 7,967), Global Youth Tobacco Survey, Vietnam, 2007

**Characteristic**	**No. (%)^a^**
**Age, y**	
13	2,531 (31.8)
14	2,948 (37.0)
15	2,488 (31.2)
**Parents' smoking status**	
Neither parent smokes	3,323 (41.7)
Either father or mother smokes	4,381 (55.0)
Both parents smoke	159 (2.0)
**Friends' smoking status**	
Do not have close friends who smoke	4,916 (61.7)
Have close friends who smoke	3,014 (37.8)
**Classes on harmful effects of smoking**	
Did not attend	1,982 (24.9)
Did attend	5,927 (74.4)
**Access to antismoking media**	
No	414 (5.2)
Yes	7,553 (94.8)
**Exposure to billboard cigarette advertising**	
No	4,965 (62.3)
Yes	3,002 (37.7)

a Totals in some sections do not equal 7,967 because of missing data.

**Table 3:** Prevalence of Smoking and Susceptibility to Smoking of Female Students (N = 7,967), Global Youth Tobacco Survey, Vietnam, 2007

**Age, y**	**Current Smoker, % (95% CI)**	**Susceptibile to Smoking^a^, % (95% CI)**
13	0.8 (0.5-1.4)	1.2 (0.7-1.8)
14	0.8 (0.5-1.3)	2.0 (1.4-2.7)
15	2.0 (1.4-2.9)	1.3 (0.8-2.0)
Overall	1.2 (0.9-1.5)	1.5 (1.2-1.9)

Abbreviation: CI, confidence interval.

a See Methods section for description of determination of participants' susceptibility to smoking.

**Table 4:** Correlates of Current Smoking Status and Susceptibility to Smoking, Female Students (N = 7,967), Global Youth Tobacco Survey, Vietnam, 2007

**Characteristic**	**Current Smoker, OR (95% CI)**	**Susceptibile to Smoking, OR (95% CI)**
**Age, y**		
13	1 [Reference]	1 [Reference]
14	0.9 (0.4-1.9)	1.4 (0.8-2.4)
15	1.8 (0.9-3.7)	0.8 (0.4-1.4)
**Girls who smoke cigarettes have more or less friends**		
Less friends or no difference	1 [Reference]	1 [Reference]
More friends	0.8 (0.4-2.0)	0.8 (0.4-1.9)
**Smoking cigarettes helps people feel more or less comfortable**		
Less comfortable or no difference	1 [Reference]	1 [Reference]
More comfortable	2.0 (1.1-3.7)	1.2 (0.8-2.0)
**Smoking cigarettes makes girls look more or less attractive**		
Less attractive or no difference	1 [Reference]	1 [Reference]
More attractive	2.4 (1.0-5.6)	2.7 (1.4-5.2)
**Knowledge of harmful effects of smoking**		
Poor	1 [Reference]	1 [Reference]
Good	0.2 (0.1-0.3)	0.4 (0.2-0.7)
**Knowledge of harmful effects of secondhand smoke**		
Poor	1 [Reference]	1 [Reference]
Good	0.5 (0.3-0.9)	0.3 (0.2-0.5)
**Parents' smoking status**		
Neither parent smokes	1 [Reference]	1 [Reference]
Either father or mother smokes	0.9 (0.5-1.5)	1.1 (0.7-1.8)
Both parents smoke	2.7 (0.9-7.7)	2.1 (0.8-6.0)
**Friends' smoking status**		
Do not have close friends who smoke	1 [Reference]	1 [Reference]
Have close friends who smoke	6.3 (3.0-13.0)	3.2 (2.1-4.8)
**Classes on harmful effects of smoking**		
Did not attend	1 [Reference]	1 [Reference]
Did attend	0.5 (0.3-0.9)	0.6 (0.4-1.1)
**Access to antismoking media**		
No	1 [Reference]	1 [Reference]
Yes	0.6 (0.3-1.4)	0.4 (0.2-0.8)
**Exposure to billboard cigarette advertising**		
No	1 [Reference]	1 [Reference]
Yes	1.2 (0.7-2.1)	1.6 (1.1-2.5)

Abbreviations: OR, odds ratio; CI, confidence interval.
